# Hepatic Sarcoidosis: An Uncommon Cause of Cirrhosis

**DOI:** 10.7759/cureus.6316

**Published:** 2019-12-06

**Authors:** Sara Ghoneim, Sherrie D Williams

**Affiliations:** 1 Internal Medicine, Case Western Reserve University MetroHealth Medical Center, Cleveland, USA; 2 Internal Medicine: Pulmonology, Case Western Reserve University MetroHealth Medical Center, Cleveland, USA

**Keywords:** epithelioid granuloma, hepatic sarcoidosis, sarcoidosis, case series

## Abstract

Sarcoidosis is a multi-organ inflammatory disease of unclear etiology. The hallmark of the disease is the formation of non-caseating granulomas. The prevalence of sarcoidosis is 5-30% in the general population and up to 80% in autopsy series. Hepatic involvement is seen in almost 50% of cases of sarcoidosis, though the clinical consequences are variable. In this study, we describe the case of three patients from our institution with hepatic sarcoidosis. Two of them eventually went on to develop liver cirrhosis.

## Introduction

Sarcoidosis is an inflammatory multi-organ disease characterized by non-caseating granulomas [1]. It has been estimated that the prevalence of the disease is 2-60 per 100,000 people worldwide [1]. In the US, the incidence of the disease is three times more in African Americans and in females with a peak age of 20-40 years [2,3]. The most common organs affected by this condition are the lymph nodes (hilar and mediastinal, 98%) followed by the lungs (90%) [3-5]. Hepatic sarcoidosis is well described in the literature and has been documented in up to 50-80% of patients diagnosed with systemic disease [2,4]. However, the clinical consequences of liver involvement are variable. Here we present a case series of three patients from our institution who were diagnosed with hepatic sarcoidosis. Two of them went on to develop cirrhosis. 

## Case presentation

Case 1

**Figure 1:**
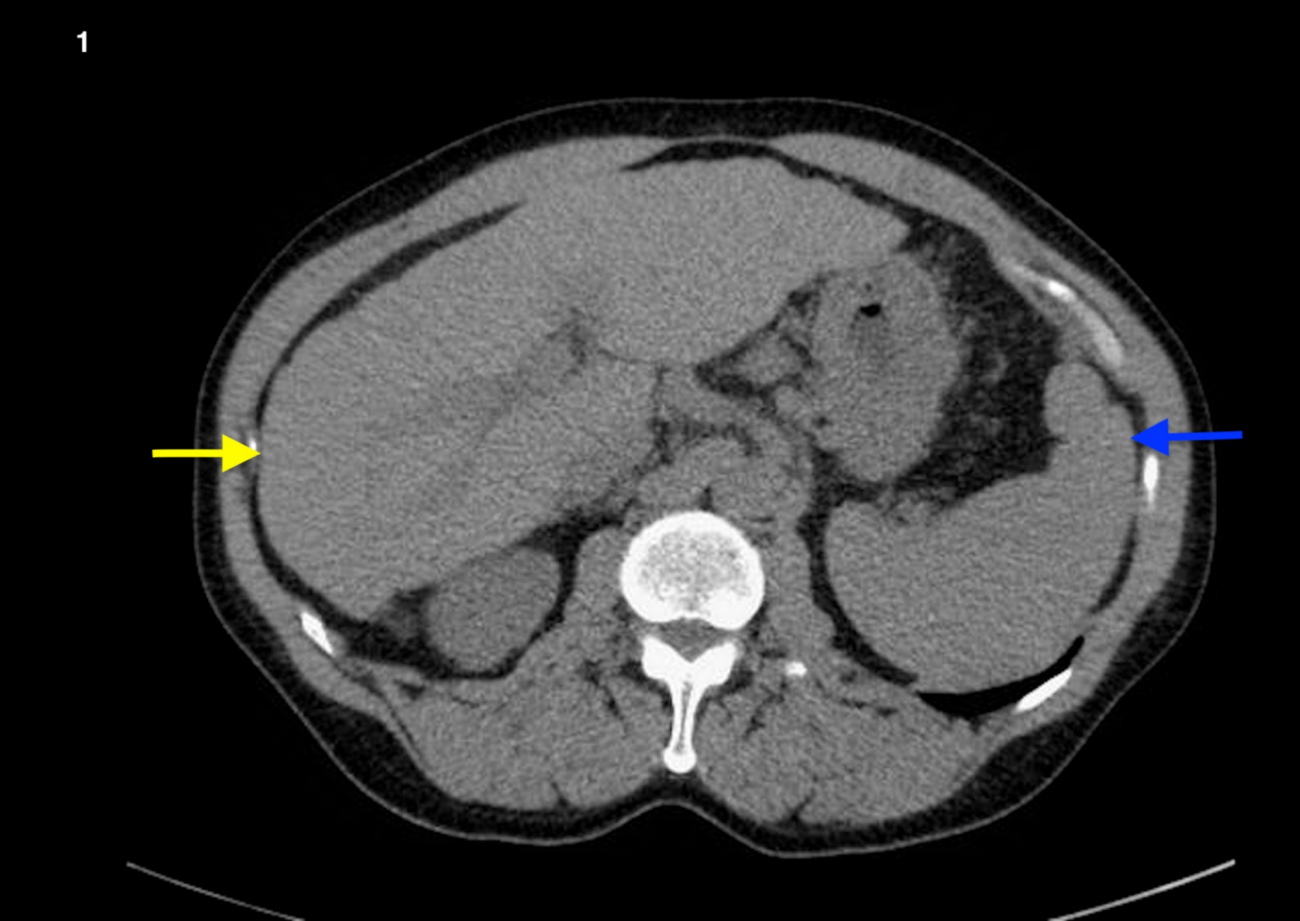
CT of the abdomen of a patient who initially presented with hepatic sarcoidosis and eventually progressed to cirrhosis A shrunken liver with irregular contours (yellow arrow) and an enlarged spleen (blue arrow)

A 59-year-old African American male with pulmonary sarcoidosis was consulted at our pulmonary clinic after he was found to have an abnormal CT of the abdomen and pelvis. Three months prior, the patient had had routine laboratory testing which had showed elevated liver enzymes [alkaline phosphatase: 604 IU/L (normal 40-200 IU/L); alanine transaminase (ALT): 63 IU/L (normal 7-40 IU/L); aspartate transaminase (AST): 81 IU/L (normal 7-40 IU/L)]. Serum gamma-glutamyltransferase (GGT) was normal. CT of the chest, abdomen, and pelvis showed nodular infiltrates in the lung. The liver and the spleen were enlarged and diffusely infiltrated with small nodular hypodensities that were concerning for lymphoma or metastatic disease (Figure 1).

The patient endorsed a three-month history of night sweats and dry cough. He had lost seven pounds. He denied hemoptysis, shortness of breath, fevers, or chills. He was a non-smoker and had never consumed alcohol. He complained of abdominal fullness and diffuse tenderness. The physical exam was otherwise normal. He had an elevated angiotensin-converting enzyme (ACE) level (165 U/L) and underwent bronchoscopy with biopsy, which confirmed a granulomatous process either infectious or sarcoid. Stains were negative for acid-fast bacilli (AFB) and fungus. Bronchoalveolar lavage was also negative for AFB and bacterial culture. The patient was diagnosed with systemic sarcoidosis and started on prednisone and methotrexate. Within six years of the diagnosis, he developed liver cirrhosis. 

Case 2

A 31-year-old African American male diagnosed with pulmonary sarcoidosis was referred to the gastroenterology clinic for abdominal pain that had started one year prior. He also complained of nausea and vomiting. He denied hematemesis, hematochezia, melena, but endorsed alternating bowel habits between constipation and diarrhea. He had lost 15 pounds in the past two months. He denied chronic alcohol use. The physical exam showed a diffusely tender abdomen. Laboratory testing was significant for elevated alkaline phosphatase (853 IU/L) and elevated aminotransaminases (AST: 103 IU/L; ALT: 185 IU/L). Serum ACE and GGT were also elevated [69 U/L, 160 IU/L (normal 3-30 IU/L for both), respectively]. An ultrasound of the liver showed an abnormal pattern of heterogeneous echogenicity of the parenchyma, which was suggestive of an infiltrative disease. CT of the liver, however, was unremarkable. He subsequently underwent an ultrasound-guided biopsy of the liver that showed non-caseating granulomatous hepatitis with bridging portal fibrosis consistent with sarcoidosis. He was started on prednisone, which resulted in the resolution of his abdominal pain and normalization of liver enzymes. 

Case 3

**Figure 2:**
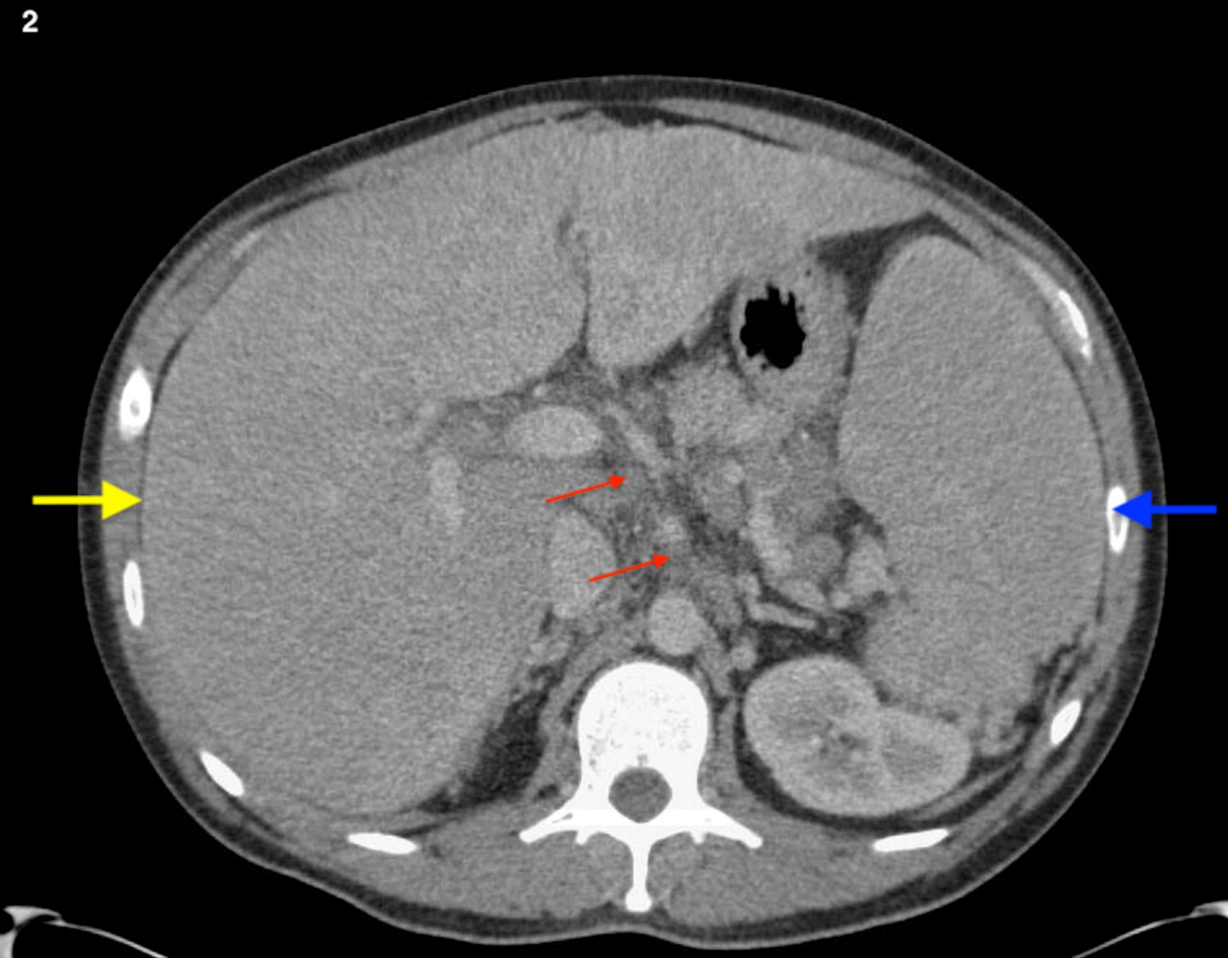
CT of the abdomen in a patient with hepatic sarcoidosis A massively enlarged liver (yellow arrow) and spleen (blue arrow) with peripancreatic and periportal lymphadenopathy (red arrow)

A 37-year-old Caucasian male with a past medical history of alcohol use disorder and pulmonary sarcoidosis was evaluated for right upper quadrant abdominal pain and distention. He endorsed a sixty-pound weight loss, jaundice, generalized fatigue, and poor appetite. Laboratory tests were significant for elevated liver enzymes (alkaline phosphatase: 615 IU/L; ALT: 31 IU/L; AST: 82 IU/L). CT of the abdomen and pelvis showed a massively enlarged liver and spleen with periportal, peripancreatic, and mesenteric lymphadenopathy (Figure 2).

Large collaterals were also seen in the upper abdomen. He underwent a liver biopsy, which showed a hepatic architecture that was disordered by broad bands of fibrosis with regenerative nodule formation consistent with cirrhosis. The portal tracts demonstrated discrete epithelioid granulomas containing multinucleated giant cells and numerous lymphocytes. Due to the presence of predominately portal based epithelioid granulomas and discrete cuffing of lymphocytes and retropharyngeal adenopathy, sarcoidosis was thought to contribute to the liver cirrhosis. The patient was started on prednisone and counseled on alcohol cessation. 

## Discussion

Sarcoidosis is an inflammatory disease of unclear etiology that can affect any organ and is seen in all parts of the world [1]. Extrapulmonary sarcoidosis is seen in 40-50% of patients [3,4]. The pathogenesis of sarcoidosis is not entirely understood but often attributed to an interplay of various factors that include gene, environment, and an individual’s immunological response [1-4]. In autopsies of patients diagnosed with systemic sarcoidosis, hepatic involvement was seen in as many as 50-80% whereas abnormal liver enzymes were only found in 10-30% [4,6]. The majority of patients with hepatic sarcoidosis are asymptomatic, with less than 20% of patients presenting with clinically significant disease [1]. There is a spectrum of clinical presentations in patients with hepatic sarcoidosis. In 50% of patients with biopsy-proven hepatic sarcoidosis, approximately 20% have palpable hepatosplenomegaly and only 10-30% have elevated liver enzymes [1]. There is also a spectrum of signs and symptoms seen in patients with hepatic sarcoidosis. Nonspecific signs and symptoms include fevers, arthralgia, and fatigue [7-9]; whereas symptoms of pruritis, jaundice, and right upper quadrant pain are more specific and suggestive of liver involvement [7-12]. Abdominal pain has been reported in 15% of patients with hepatic sarcoidosis and jaundice has been observed in less than 5% of the patients [7,10]. Portal hypertension can occur in long-standing liver disease, and this could be ascribed to the compression of the portal venules by granulomas. Approximately 0.012% of patients with liver sarcoidosis develop end-stage liver disease that requires transplantation [5,6,12-14]. Unfortunately, these patients have a worse prognosis than those with other liver diseases and recurrence has been documented in transplanted patients [9].

Several mechanisms have been proposed about the ways in which sarcoidosis causes injury to the liver [6-8]. Granuloma formation with subsequent inflammation and fibrosis can lead to cirrhosis. Ascites and systemic disease generally ensue [5-8]. Evaluation by serum chemistries and liver enzymes is essential in identifying the extent of the disease [13,14]. Elevations in alkaline phosphatase and/or GGT generally correlate with cholestasis [13,14]. Hepatobiliary alkaline phosphatase can be as high as 5-10 as the upper normal limit, while aminotransferase elevations are generally milder, as was seen in all of these cases. Serum ACE is elevated in 60% of the cases of sarcoidosis [15]. However, this test is considered to have poor sensitivity and specificity [15]. 

Often, ultrasonography, MRI, or CT may show hepatomegaly or multiple hypodense lesions which can be confused with metastatic disease [16]. Transient elastography (TE) is a non-invasive imaging modality that measures the degree of liver stiffness using an ultrasound probe [16]. This tool is easy to use with good reliability but is associated with high interobserver variability. In addition, several scoring systems that rely on serum markers can be used to evaluate the extent of liver disease. They are non-invasive and cost-effective. For example, the AST-to-platelet ratio index (APRI) has a sensitivity of 53% and specificity of 93% for diagnosing and monitoring liver fibrosis. Similarly, an AST-to-ALT ratio greater than one in non-alcoholic liver disease is suggestive of cirrhosis with a modest sensitivity and specificity values (51% and 71%, respectively). FibroSURE™ (Laboratory Corporation of America, Raritan, NJ) is a commercial serological test that takes into account direct serum markers of fibrosis in addition to the patient's age and gender [16]. Unfortunately, these scoring systems have primarily been studied in patients with hepatitis B and C, and their effectiveness as screening tools in hepatic sarcoidosis is unknown [17]. Liver biopsy is recommended for patients with moderate-to-severe liver-test findings, especially when a competing etiology is present.

Management of patients with hepatic sarcoidosis could involve observation without pharmacological intervention when the liver’s synthetic function is normal with no evidence of cholestasis [6,9]. Medical management is necessary when there is laboratory evidence of cholestatic disease and in patients with comorbidities who are at high risk of developing serious liver disease. Steroids have been shown to reduce granuloma and liver size by reducing the associated inflammation [5,18]. Generally, treatment duration is one year before tapering is initiated [18]. In addition, methotrexate has been shown to be of benefit in patients with hepatic sarcoidosis. Ursodeoxycholic acid is helpful in patients with intrahepatic cholestasis [9,18]. The mortality rate from sarcoidosis is estimated to be between 1-5%, with death occurring due to severe impairment of pulmonary and cardiac functions or the central nervous system rather than hepatic causes [6,9]. 

## Conclusions

We presented three cases of hepatic sarcoidosis with similar clinical findings. Two of the patients eventually went on to develop liver cirrhosis. Hepatic sarcoidosis should always be suspected in patients with systemic sarcoid. The implications of these findings, however, remain ambiguous as seen in our case series, with one patient showing improvement on steroid use while others progressing to cirrhosis in the setting of alcohol use. As we gain more insight into the various aspects of sarcoidosis, it would be interesting to identify and focus on the subset of patients who present exclusively with localized liver disease. This would help us to better elucidate the pathophysiological pathways that lead to one phenotype versus the other. 
